# Association between early ambulation exercise and short-term postoperative recovery after open transforaminal lumbar interbody fusion: a single center retrospective analysis

**DOI:** 10.1186/s12891-023-06395-w

**Published:** 2023-05-04

**Authors:** Jingwen Liao, Zhou Qi, Biying Chen, Purun Lei

**Affiliations:** 1 Department of Bone Surgery, The Third Affiliated Hospital, Sun Yat-Sen University, Guangzhou, China; 2 Department of Gastrointestinal Surgery, The Third Affiliated Hospital, Sun Yat-Sen University, Guangzhou, China

**Keywords:** Early ambulation, Time interval, Transforaminal lumbar interbody fusion, Length of stay

## Abstract

**Background:**

Early ambulation in patients undergoing transforaminal lumbar interbody fusion (TLIF) surgery is recommended, however, the precise time interval after open surgery has never been specified. Current retrospective analysis was conducted aiming to clarify an accurate time interval.

**Methods:**

A retrospective analysis of eligible patients was conducted using the databases of the Bone Surgery Department, Third Affiliated Hospital of Sun Yat-sen University from 2016 to 2021. Data pertaining to postoperative hospital stay length, expenses, incidence of complications were extracted and compared using Pearson’s χ2 or Student’s t-tests. A multivariate linear regression model was conducted to identify the relationship between length of hospital stay (LOS) and other outcomes of interest. A propensity analysis was conducted to minimize bias and to evaluate the reliability of results.

**Results:**

A total of 303 patients met the criteria and were included for the data analysis. Multivariate linear regression results demonstrated that a high ASA grade (*p* = 0.016), increased blood loss (*p* = 0.003), cardiac disease (*p* < 0.001), occurrence of postoperative complications(*p* < 0.001) and longer ambulatory interval (*p* < 0.001) was significantly associated with an increased LOS. The cut-off analysis manifested that patients should start mobilization within 3 days after open TLIF surgery (B = 2.843, [1.395–4.292], *p* = 0.0001). Further comparative analysis indicated that patients who start ambulatory exercise within 3 days have shorter LOS (8.52 ± 3.28d vs 12.24 ± 5.88d, *p* < 0.001), total expenses ( 9398.12 ± 2790.82vs 10701.03 ± 2994.03 [USD], *p* = 0.002). Propensity analysis revealed such superiority was stable along with lower incidence of postoperative complications (2/61 vs 8/61, *p* = 0.0048).

**Conclusions:**

The current analysis suggested that ambulatory exercise within 3 days for patients who underwent open TLIF surgery was significantly associated with reduced LOS, total hospital expenses, and postoperative complications. Further causal relationship would be confirmed by future randomized controlled trials.

## Introduction

Back pain and spinal disorders are one of the most common medical problems worldwide. A lumbar spinal fusion may ameliorate symptoms and help to improve patients’ quality of life. Patients were encouraged to initiate ambulatory exercise early after surgery according to the ERAS (Enhanced Recovery After Surgery) protocol [1–3]. Several studies have investigated the benefits of early ambulation in patients who underwent spine surgeries [4, 5]. Prolonged immobilization after surgery has been shown to have deleterious effects on pulmonary function, muscles, urinary tract, and skin integrity; it can also increase the risk of various complications, such as deep venous thrombosis (DVT), pulmonary embolism, pulmonary infection, and urinary tract infection, as well as prolong the length of hospital stay (LOS) [6].

Despite detailed preoperative and postoperative education was conducted, patients may still avoid ambulation for a few days after surgery due to individual preference, wound pain, and residual symptoms [7]. Due to such “deliberate postpone”, an specified time interval to start ambulatory exercises after spinal surgery was in great needed [8, 9]. Herein, we retrospectively analyzed the effects of early mobilization on LOS and postoperative complications in patients who underwent transforaminal lumbar interbody fusion (TLIF) surgery and explored the ideal time needed for ambulatory rehabilitation after surgery, which was never reported elsewhere before.

## Methods

### Study population

This retrospective study was approved by the Ethics Committee. Eligible patients were identified by searching the database of the Bone Surgery Department from 2016 to 2021. All patients have signed the informed consent of perioperative data analysis after hospitalization.

Patient inclusion criteria were as follows: 1. Underwent elective open TLIF surgery; 2. Baseline characteristics and operative information were available; 3. No more than 2 segments fusion 3. Time interval between surgery and ambulation training was traceable. Patient exclusion criteria were as follows: 1. Patient who underwent emergency surgeries; 2. Patients underwent minimal invasive TLIF surgery; 3. Insufficient data for analysis; and 4. Patients with severe health conditions or nerve compression symptoms affecting daily activities: such as severe COPD, paralysis or gravis myasthenia, cauda equina syndrome, etc.

### Outcomes

The following demographic and clinical information were extracted from the database: age, sex, body mass index (BMI), co-morbidities, ASA score, operative duration, estimated blood loss (EBL), time interval between surgery and ambulation, surgical wound length, preoperative hemoglobin and serum albumin level. The outcomes of interest were length of hospital stay (LOS), expense, and rates of postoperative complications, which included surgical site infection, respiratory/urinary infection, cerebrospinal fluid leakage, and DVT.

### Surgical procedure

All surgeries were performed by same surgeon and nursing team under general anesthesia. Two grams of cefixime was administered intravenously 30 min before the surgery. Thereafter, the patient was placed in the prone position, and surgical procedures were initiated after proper sterilization. The preoperative planned level is determined by C-arm fluoroscopy. A standard midline incision was made; after dissection, the paravertebral muscles were carefully retracted to reveal the transverse processes. Polyaxial pedicle screws were placed on both sides at the specified levels, and distraction were performed with specially prepared retractors. Thus, instead of performing a laminectomy, we have an adequate field of view and application with the inferior facet joint resection of the upper segment of the facet joint. A gentle retraction was made with the dura retractor to perform root and dura decompression. The disc level was reached between the root and the dura, and the disc content was carefully excised. The upper and lower end plates were excised with a curette. The disc and the end plate residues were completely removed by washing them thoroughly with water. The resulting grafts were placed in the disc space and the TLIF cage was placed appropriately. Compression and fixation were provided with the help of rods. Finally, a drain was placed under the skin, and the skin above the drain was closed.

### Rehabilitation

The brace was manufactured before the surgery and applied. Drainage is usually complete within 48 h after the surgery, or the total volume is less than 50 ml per day. Prophylactic antibiotics were routinely administered until 48 h after the surgery. Intravenous nonsteroidal anti-inflammatory drug was applied every 12 h postoperatively. The urinary catheter was removed on postoperative day one, and oral intake resumed 6 h after the surgery. The patient’s rehabilitation plan was made according to individual factors and clinical judgments by the surgeon and a rehabilitation therapist, under the following principles postoperatively:1. Residual symptom and pain evaluation twice a day; 2. muscle force and tone measurement every morning; 3. lower proximal limbs horizontal movement and muscle contraction exercise while in the supine position; 4. straight leg raises exercise (one leg straight and one knee bent alternately) in sitting up position; 5. Air pressure massage treatment twice a day; 6. Ambulatory evaluation. All patients were encouraged to initiate mobilization with the help of walking aid and brace helmet at the earliest, but the time of the patient’s decision depended on themselves. Routinely, patients were discharged after removal of the drain and when the wound healed without complications.

### Statistical analysis

Categorical variables are presented as frequencies and continuous variables are presented as means ± standard deviation. Pearson’s χ2 or Fisher’s exact tests were used to analyze the categorical variables. Student’s t-tests were used for analyzing normally distributed data; else, Mann–Whitney U tests were used to analyze continuous variables. A multivariate linear regression model was used to identify the relationship between the length of hospital stay and other factors. Based on the assumption that delayed ambulatory exercise was associated with longer hospital stay, ambulatory time interval was assessed as a categorical variable by another multivariate linear regression model after adjusting for other potential confounders (i.e., sex, age, stage, ASA, BMI, anemia status, lower serum albumin, surgical duration and comorbidities) to reveal the significance of each time interval. All data analyses were performed with SPSS v22 (Armonk, NY: IBM Corp), and *P* < 0.05 was considered to indicate statistical significance.

## Results

### Patient characteristics

**Fig. 1:**
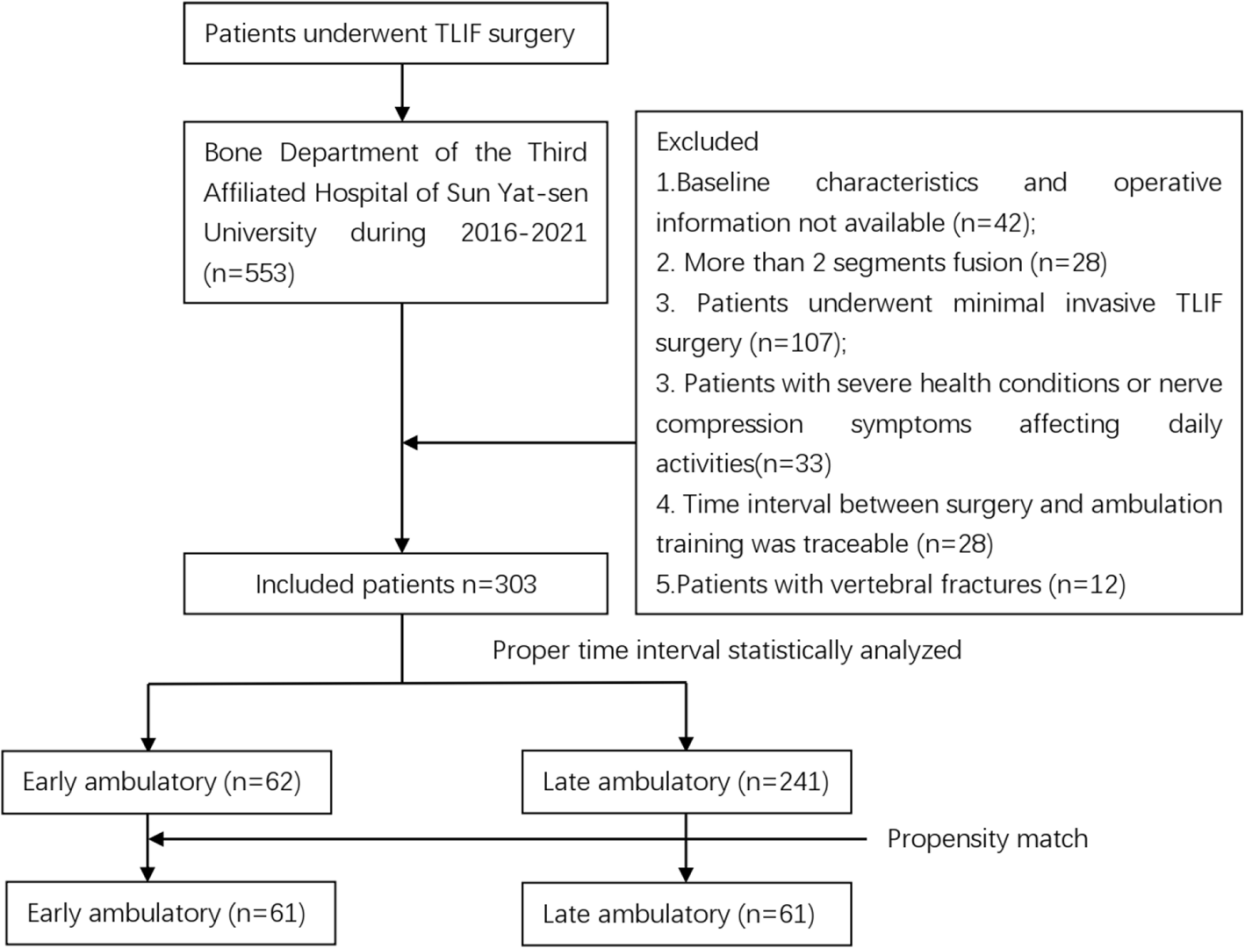
Flow chart for patient selection in the current study

In total, 303 patients were included for the data analysis (156 men and 147 women). The mean age was 57.60 ± 13.55 years, and mean BMI was 23.66 ± 3.92 kg/m^2^. Most patients had lumbar spinal stenosis (200/303) and lumbar disc herniation (75/303); other reasons for surgery were lumbar instability (28/303). The time interval between surgery and first ambulatory exercise ranged from 1–20 days, with a median of 5 days. The average LOS was 11.47 ± 5.64 days. Baseline characteristics are shown in Table 1 and patients selection procedure are shown in Fig. 1.

**Table 1:** Baseline characteristics of all patients enrolled, *BMI*: Body Mass Index; *ASA*: American Society of Anesthesiologists, *VAS*: visual analogue scale [10]; *ADL*: Activity of Daily Living Scale [11], *ODI*: The Oswestry Disability Index [12], *USD*: US Dollar

Items	value
Age	57.60 ± 13.55
Sex (male/female)	156/147
BMI ( kg/m^2^)	23.66 ± 3.92
ASA grade (1/2/3)	153/124/26
Hemoglobin (preop) (g/L)	133.95 ± 17.97
Albumin (preop) (g/L)	41.25 ± 4.06
Surgical duration (min)	191.02 ± 50.19
Blood Loss (ml)	211.98 ± 121.42
Wound length (cm)	8.82 ± 2.87
Ambulatory time interval (d)	5.39 ± 2.65
LOS (d)	11.47 ± 5.64
Total expenses [USD]	10,434.43 ± 2995.71
Commodities	
Hypertension	75
Diabetes	23
Cardiac coronary disease	7
ODI scale (preop)	49.42 ± 8.71
ADL scale (preop)	58.40 ± 6.73
VAS value (preop)	6.01 ± 1.41
Primary Disease	
spinal stenosis	200
lumbar disc herniation	75
lumbar instability	28

### Cut-off Point analysis for ambulation and LOS

A multivariate linear regression model was utilized to identify the relationship between the length of hospital stay and individual characteristics (sex, age, and BMI), preoperative hemoglobin, serum albumin level, surgical time, estimated blood loss, ASA grade, and postoperative complications, and a Enter method was used for variable selection. The results of regression analysis indicated that ASA grade, EBL, cardiac disease, occurrence of postoperative complications and ambulatory interval were significantly associated with LOS. Multiple correlation coefficient R value and adjusted R2 value for the model were 0.664 and 0.414 respectively, demonstrating a higher correlation between results and all included variables. ANOVA evaluation significance of the model was < 0.001, manifesting a clear linear relationship. The results proved that patients had longer LOS when ambulation was initiated later after the surgery (Table 2).

**Table 2:** Relationship between all variables and LOS by multivariate linear regression analysis, *BMI*: Body Mass Index; *ASA*: American Society of Anesthesiologists; *EBL*: estimate blood loss

	B	Std. Error	standardized coefficients	Significance	Lower CI	Upper CI
Constant value	0.736	2.233		0.742	-3.658	5.131
Sex (Female)	0.023	0.552	0.002	0.967	-1.064	1.110
Age	0.033	0.021	0.080	0.108	-0.007	0.074
BMI	-0.037	0.072	-0.026	0.605	-0.178	0.104
ASA grade	1.046	0.432	0.120	**0.016**	0.196	1.896
Hemoglobin	-0.689	0.932	-.035	0.461	-2.523	1.146
Serum albumin	0.144	1.110	0.006	0.897	-2.042	2.329
Surgical duration	0.006	0.006	0.058	0.246	-0.004	0.017
Blood loss	0.006	0.002	0.138	**0.003**	0.002	0.011
Surgical length	-.029	0.096	-0.015	0.760	-0.218	0.160
Ambulatory time	1.036	0.103	0.486	**0.000**	0.834	1.238
Hypertension	-1.052	0.660	-0.081	0.112	-2.352	0.247
Diabetes	-1.477	0.981	-0.069	0.133	-3.408	0.455
Coronary disease	4.686	1.708	0.125	**0.006**	1.325	8.048
Postoperative complications	4.394	0.909	0.226	**0.000**	2.606	6.182

The cut-off value was then estimated, while the ambulatory interval was treated as categorical data for a different threshold. We found that when the time to ambulation was ≥ 4 day, LOS was significantly prolonged, indicating that the appropriate time interval should be within 3 days (Table 3).

**Table 3:** Relationship between time to ambulation and LOS by multivariate linear regression analysis

Time interval	B	Std. Error	standardized coefficients	*P* value	Lower CI	Upper CI
2 days	2.564	2.964	0.045	0.388	-3.269	8.397
3 days	1.420	1.287	0.058	0.271	-1.114	3.953
4 days	2.843	0.736	0.203	0.0001	1.395	4.292
5 days	3.292	0.599	0.284	< 0.0001	2.114	4.471
6 days	3.830	0.600	0.328	< 0.0001	2.648	5.011

### Comparison and propensity analysis

To further analyze the potential advantage of the threshold, patients were divided into “early ambulatory (≤ 3 days)” and “late ambulatory (≥ 4 days)” groups according to the time interval between first ambulatory exercise and TLIF operation. A total of 62 patients was categorized into the early ambulatory group and 241 patients in the late ambulatory group. The results indicated that patients from the “early ambulatory group” had shorter LOS and total expenses as shown in Table 4.

**Table 4:** Baseline features and postoperative outcomes after groups were defined, *BMI*: Body Mass Index; *ASA*: American Society of Anesthesiologists, *VAS*: visual analogue scale [10]; *ADL*: Activity of Daily Living Scale [11], *ODI*: The Oswestry Disability Index [12], *LOS*: length of stay; *EBL*: estimate blood loss; *USD*: US dollar; *SSI*: surgical site infection, *CSF*: cerebrospinal fluid

	Early ambulatory (*n* = 62)	Late ambulatory (*n* = 241)	*P* value
Gender (male/female)	37/25	119/122	0.148
Age (year)	52.45 ± 13.48	58.92 ± 13.28	**0.0003**
BMI (kg/m^2^)	23.21 ± 3.49	23.90 ± 3.90	0.209
ASA (1/2/3)	39/16/7	114/108/19	**0.024**
ODI (preop)	48.92 ± 8.31	49.83 ± 8.99	0.471
VAS (preop)	6.02 ± 1.45	6.00 ± 1.41	0.936
ADL (preop)	60.73 ± 6.96	62.06 ± 7.25	0.193
Comorbidity			
Hypertension	10	65	0.078
Diabetes mellitus	5	18	0.873
Heart disease	2	5	0.945
Preop hemoglobin (g/L)	134.35 ± 17.80	132.73 ± 21.70	0.587
Preop Albumin (g/L)	41.80 ± 4.22	41.11 ± 4.02	0.235
Disease			
spinal stenosis	37	163	0.238
lumbar disc herniation	19	56	0.228
lumbar instability	6	22	0.89
Surgical duration (min)	184.90 ± 50.13	192.59 ± 49.93	0.283
EBL (ml)	224.35 ± 123.18	208.80 ± 121.02	0.514
Wound length (cm)	9.21 ± 2.88	8.80 ± 2.88	0.369
Ambulatory interval (d)	2.67 ± 0.57	6.09 ± 2.52	** < 0.001**
LOS (d)	8.52 ± 3.28	12.24 ± 5.88	** < 0.001**
Total expenses [USD]	9398.12 ± 2790.82	10,701.03 ± 2994.03	**0.002**
Overall complications	2	26	0.067
Pneumonia	0	6	0.45
Reoperation	1	0	1
SSIs	1	13	0.355
CSF leakage	0	4	0.129
Urinary infection	0	3	1

The current data demonstrated that early ambulatory exercise is associated with lower risk of postoperative complications. To further clarify the relationship, propensity matched analysis with 1:1 ratio was conducted to minimize the bias in baseline characteristic differences, including age, ASA grade, sex, primary disease, and surgical features. A total of 122 patients were considered in the data analysis after balancing the differences in age, sex, ASA, BMI, primary disease, preoperative hemoglobin, and serum albumin. As shown in Table 5, the baseline features were comparable between both groups. Furthermore, the results of propensity matched analysis revealed that LOS was shorter in the early ambulatory group with lower overall complications and total expenses.

**Table 5:** Baseline features and postoperative outcomes of propensity matched analysis, *BMI*: Body Mass Index; *ASA*: American Society of Anesthesiologists, *VAS*: visual analogue scale [10]; *ADL*: Activity of Daily Living Scale [11], *ODI*: The Oswestry Disability Index [12], *LOS*: length of stay; *EBL*: estimate blood loss; *USD: US dollar*; *SSI*: surgical site infection, *CSF*: cerebrospinal fluid

	Early ambulatory (*n* = 61)	Late ambulatory (*n* = 61)	*P* value
Gender (male/female)	36/25	35/26	0.854
Age (year)	52.51 ± 13.59	54.10 ± 13.90	0.524
BMI (kg/m^2^)	22.75 ± 3.90	23.50 ± 4.04	0.303
ASA (1/2/3)	38/16/7	36/20/5	0.660
ODI (preop)	48.85 ± 8.34	48.85 ± 8.75	1
VAS (preop)	5.98 ± 1.44	6.01 ± 1.31	0.948
ADL (preop)	58.69 ± 7.07	58.11 ± 6.72	0.647
Comorbidity			
Hypertension	10	12	0.637
Diabetes mellitus	5	4	1
Heart disease	2	1	0.874
Preop hemoglobin (g/L)	134.08 ± 17.82	134.85 ± 19.32	0.819
Preop Albumin(g/L)	41.98 ± 4.00	41.33 ± 4.46	0.398
Disease			
spinal stenosis	37	39	0.709
lumbar disc herniation	18	17	0.841
lumbar instability	6	5	0.752
Surgical duration (min)	185.48 ± 51.36	185.27 ± 45.63	0.982
EBL (ml)	224.75 ± 124.16	191.23 ± 103.04	0.107
Wound length (cm)	9.00 ± 2.84	8.43 ± 2.44	0.234
Ambulatory interval (d)	2.67 ± 0.57	6.41 ± 3.49	**0.0001**
LOS (d)	8.95 ± 4.36	12.70 ± 6.13	**0.0001**
Total expenses [USD]	9409.39 ± 2812.55	10,481.33 ± 3049.43	**0.048**
Overall complications	2	8	**0.048**
Pneumonia	0	3	0.242
Reoperation	1	0	1
SSIs	1	3	0.611
CSF leakage	0	1	1
Urinary infection	0	1	1

## Discussion

Spine surgical interventions have evolved during the past decades, and clinical research has mainly focused on innovation of surgical approach and techniques [5]. However, spine surgery is notorious for its burdensome recovery process [13, 14]. With opportunities for progress, combined with the benefits of ERAS in other specialties such as abdominal and orthopedic surgery [2, 3, 15], the field of spine surgery is prepared for an evolution toward novel perioperative care.

Efforts on ERAS in the spine surgical field have emerged in the past few years. Ali [4], and Wainwright [5] investigated the implications and feasibility of implementing a spine ERAS program. Wang [14], Gornitzky [16], and Muhly [17] explored other spinal ERAS projects, including endoscopic lumbar fusions and correction of scoliosis. Porche reported ERAS was associated with decreased operative time, reduced length of stay, decrease in IV opioid consumption, and improved physiological outcomes for open 1- and 2-level TLIF [18, 19]. All these trials demonstrated reduced length of stay and acute care costs without increase in re-admission rates.

All ERAS principles in other fields recommend early mobility, usually defined as the patient getting out of bed as soon as possible after surgery. Early ambulation has been proven to be associated with a shorter LOS, lower rates of urinary tract infections, pulmonary complications, and thromboembolism [1–3, 6, 8, 9]. Hurdles that delay ambulation after spine surgeries include rehabilitation procedure, patient symptoms, and medical device restriction. All patients should undergo systematic on-bed training and evaluation by a rehabilitation therapist before mobilization. Symptoms such as weakness and generalized fatigue may prevent elderly patients from ambulation. Additionally, the lack of staff and availability of assistive devices also delay ambulation. Finally, medical devices such as intravenous lines and catheters also restrain recovery. Thus, despite the known benefits of early mobility and the many protocols cited for critical care patients, there is little guidance available on how soon after a spine surgery should patients get out of bed and walk [20]. In the current study, patients with severe nerve compression symptoms (paralysis, cauda equina syndrome, etc.) were excluded due to the longer interval between surgery and ambulatory training. Preoperative VAS score [10], activities of daily living (ADL) [11], and Oswestry Disability Index (ODI) score [12] were comparable between both the early and late ambulatory groups, indicating minimal symptomatic disequilibrium among patients. Furthermore, systematic preoperative education on the necessity of early mobility and engagement in postoperative care is conducted routinely in our center. This procedure helps patients and hospital staff accomplish early rehabilitation.

Since postoperative LOS was a continuous variable, binary logistic regression was inappropriate to explore its independent risk factors. Thus, association between LOS and other variables, including both continuous and categorized variables, were analyzed using multivariate linear regression. Initially, the mobilization interval was estimated as a continuous variable. The results displayed that delayed ambulation, coronary artery disease, higher ASA grade, larger EBL, and the occurrence of postoperative complications were significantly associated with longer LOS. In addition, there was no multicollinearity across all variables, indicating that the internal correlation had no impact on linear regression.

To further clarify the appropriate cut-off time, the time interval between starting ambulation after surgery was analyzed as a categorical variable according to specific days. Multivariate linear regression results revealed that postoperative LOS was significantly positively correlated with the interval of 4 days or more after adjusting for other confounding factors, m. However, there was no clear linear relationship between postoperative LOS and time intervals of 3 days or fewer. These results indicated the ambulatory time should start at least 3 days after surgery to minimize its influence on the LOS. Patients were then divided into different groups with a cut-off point of 3 days to ambulation to verify this finding. The early ambulatory group (≤ 3 days) had a significantly shorter LOS, and furthermore, lower total expenses and overall complications such as the occurrence of surgical site infection.

There are some explanations for this result. First, early ambulatory training could reduce incidence of postoperative complications such as hypostatic pneumonia, deep vein thrombosis, postural hypotension, sores, and pulmonary embolism, thereby reducing LOS [21–25]. Second, upright mobility encourages lumbar muscle contraction which aids the patient’s functional recovery [26, 27]. Mobility also helps the hematoma drainage thus relieving residual symptoms. Third, early ambulation accelerates gastrointestinal tract peristalsis thus preventing the discomfort of constipation, nausea, and vomiting, encouraging eating, and decreasing reluctance to leave the bed [28]. Finally, early rehabilitation increases patient confidence of treatment success which results in earlier discharge from the hospital.

However, there were some biases in baseline characteristics, such as age, ASA grade, and other variables, particularly between the early and delayed exercise groups. Patients who regained mobility earlier were much younger and had lower ASA grade. Therefore, a 1:1 ratio of propensity score matching was conducted based on age, gender, ASA grade, BMI, primary disease, preoperative hemoglobin, and serum albumin. Similar patient characteristics was observed between both groups after match, eliminating fluctuations in baseline variables that may compromise the results. Data analysis confirmed the superiority of early ambulatory training in reducing LOS and total expense. Furthermore, occurrence of overall complications also decreased significantly in the early mobilization group. Another bias was the conservative postoperative rehabilitation protocol in our institution during the study. Mobilization was initiated at the patient's discretion rather than following an ERAS protocol that encourages patients to start mobilization on first postoperative day. Patients were prone to delaying ambulation due to individual preference, wound pain, and residual symptoms and result in some selection bias. Based on current study, we are conducting a prospective clinical trial on the implementation of the concessive rehabilitation program for patients underwent open TLIF operations. Recruited patients were asked to wear electronic pedometers and encouraged to accomplish a predefined step goals within 3 days postoperatively. A comparison was made between perioperative results and patients' satisfaction with those who began ambulation strictly according to ERAS protocol. Current study offers an alternative rehabilitation strategy for patients undergoing open TLIF surgery. Clinical feasibility and patient satisfaction of the modified rehabilitation plan will be elucidated in our future research.

Our methodology has several strengths. The detailed database provided us with sufficient sample size to analyze the association between time to ambulation and outcomes of interest. The detailed “early threshold” of significant advantage in recovery and safety was first reported and analyzed in this study. Furthermore, propensity analysis helped minimize bias in baseline characteristics of enrolled patients thereby enhancing the generalizability of our findings.

However, out study has several limitations. First, this was a single-center analysis, which limits the external validity of our findings. Second, although the ambulatory modes were examined by reviewing the database and medical records, the compliance and quality could not be evaluated. Third, as reported in other retrospective studies, historical bias may still exist despite propensity analysis. Future work should therefore examine the relationship between ambulatory time and recovery using more restricted inclusion criteria. We are also looking forward to future well designed randomized controlled trials proving the causal relationship.

## Conclusions

The current analysis suggested that ambulatory exercise within 3 days for patients who underwent open TLIF surgery was significantly associated with reduced LOS, total hospital expenses, and postoperative complications. Further causal relationship should be confirmed by future randomized controlled trials.

## Data Availability

Eligible patients were identified by searching the database of the Bone Surgery Department from 2016 to 2021. We cannot provide detailed data file due to ethical restriction in privacy, readers can contact me through email in leipurun@163.com if needed.
